# Removal of Mannose-Ending Glycan at Asn^2118^ Abrogates FVIII Presentation by Human Monocyte-Derived Dendritic Cells

**DOI:** 10.3389/fimmu.2020.00393

**Published:** 2020-03-26

**Authors:** Sandrine Delignat, Julie Rayes, Suryasarathi Dasgupta, Bagirath Gangadharan, Cécile V. Denis, Olivier D. Christophe, Jagadeesh Bayry, Srinivas V. Kaveri, Sébastien Lacroix-Desmazes

**Affiliations:** ^1^Institut National de la Santé et de la Recherche Médicale, Centre de Recherche des Cordeliers, Sorbonne Université, Université de Paris, Paris, France; ^2^HITh, UMR_S1176, INSERM, Université Paris-Saclay, Le Kremlin-Bicêtre, France

**Keywords:** hemophilia A, factor VIII, FVIII inhibitors, N-glycosylations, immunogenicity of therapeutic proteins

## Abstract

The development of an immune response against therapeutic factor VIII is the major complication in hemophilia A patients. Oligomannose carbohydrates at N239 and/or N2118 on factor VIII allow its binding to the macrophage mannose receptor expressed on human dendritic cells, thereby leading to factor VIII endocytosis and presentation to CD4+ T lymphocytes. Here, we investigated whether altering the interaction of factor VIII with mannose-sensitive receptors on antigen-presenting cells may be a strategy to reduce factor VIII immunogenicity. Gene transfer experiments in factor VIII-deficient mice indicated that N239Q and/or N2118Q factor VIII mutants have similar specific activities as compared to non-mutated factor VIII; N239Q/N2118Q mutant corrected blood loss upon tail clip. Production of the corresponding recombinant FVIII mutants or light chains indicated that removal of the N-linked glycosylation site at N2118 is sufficient to abrogate *in vitro* the activation of FVIII-specific CD4+ T cells by human monocyte-derived dendritic cells. However, removal of mannose-ending glycans at N2118 did not alter factor VIII endocytosis and presentation to CD4+ T cells by mouse antigen-presenting cells. In agreement with this, the N2118Q mutation did not reduce factor VIII immunogenicity in factor VIII-deficient mice. Our results highlight differences in the endocytic pathways between human and mouse dendritic cell subsets, and dissimilarities in tissue distribution and function of endocytic receptors such as CD206 in both species. Further investigations in preclinical models of hemophilia A closer to humans are needed to decipher the exact role of mannose-ending glycans in factor VIII immunogenicity.

## Introduction

Five to thirty percent of patients with hemophilia A develop inhibitory anti-factor VIII (FVIII) antibodies following replacement therapy with therapeutic FVIII (1). The reasons for the elevated immunogenicity of therapeutic FVIII, as compared to other therapeutic glycoproteins, have been intensively investigated. These include congenital (2, 3) or bleeding-induced (4) chronic inflammation favoring the recruitment and activation of antigen-presenting cells (APCs) and of immune effectors, and disequilibrium in regulatory elements of the humoral or cellular arms of the adaptive immune system (5–7). A mandatory step for developing an anti-FVIII immune response is the endocytosis of the exogenously administered FVIII by APCs, such as dendritic cells (DCs) and macrophages, and the presentation of FVIII-derived peptides on major histocompatibility complex class II (MHC II) molecules by APCs to CD4+ T lymphocytes (8, 9).

These last decades, we and others have explored the mechanisms by which FVIII is endocytosed by APCs either by targeting endocytic receptors (10, 11) or by masking and mutating specific amino acid residues in FVIII (11–13). While there is evidence that residues in the C1 (R2090, K2092, and F2093) (12) and C2 (R2215 and R2220) (13) domains of FVIII are involved in the endocytosis of FVIII by human monocyte-derived DCs (MO-DCs), the nature of the involved endocytic receptor(s) remains unknown. *In vitro* experiments using an excess of mannan demonstrated the importance of mannose-sensitive receptors for the endocytosis of different FVIII products by human DCs and for the ensuing presentation of FVIII-derived peptides to T cells (10, 14). FVIII is a heterodimeric glycoprotein composed of a heavy chain (A1-a1-A2-a2-B domain) and a light chain (a3-A3-C1-C2 domain) linked by non-covalent binding. FVIII contains 20 N-glycosylations that are unequally distributed over the FVIII molecule: two on the A1 domain, one on the A3 and C1 domains and the remaining on the B domain (15). Both plasma-derived FVIII, recombinant full-length (FL) and B domain-deleted FVIII (BDD-FVIII) have been reported to contain mannose-ending glycans at positions N239 and N2118 of the A1 and C1 domain, respectively (16, 17).

Interestingly, both pre-incubation of DCs with an antibody toward the macrophage mannose receptor (CD206), and enzymatic removal of mannosylated glycans on FVIII, lead to reduced FVIII presentation to a human CD4 + T cell line (10). Conversely, a recombinant CD206 construct was shown to bind both the light and heavy chains of BDD-FVIII. Recombinant FL-FVIII and BDD-FVIII products, commercially available at the time of the studies, interact with CD206 (10, 18). While mannose-ending glycans on foreign glycoproteins generally mediate pathogens recognition and elimination by the immune system, oligomannose carbohydrates on self-antigens and their binding to CD206 have been implicated in their catabolism (19). Here, we studied the involvement of the two mannose-ending glycans present at positions N239 and N2118 of FVIII in its immunogenicity *in vitro* and *in vivo*.

## Materials and Methods

### Animals

Seven to 12 week-old FVIII exon 16 knock-out C57Bl/6 mice (from Prof. H. H. Kazazian, University of Pennsylvania School of Medicine, Philadelphia) (20) and double von Willebrand factor (VWF)/FVIII deficient mice were used. SureL1 mice are HLA-A2.1-/HLA-DR1-transgenic, H-2 class I-/class II-knockout mice (from Dr. Yu-Chun Lone, INSERM, Villejuif, France) (21). The experimental procedures were approved by the local ethics committee (Charles Darwin N°5, Paris, France), and accreditations have been obtained from the French government (authorization #2058.04).

### Cloning of Wild-Type and Mutant B Domain-Deleted FVIII for *in vivo* Gene Transfer

All clonings and generation of FVIII variants were performed using a BDD-FVIII coding sequence. Indeed, both FL-FVIII and BDD-FVIII demonstrate similar levels of immunogenicity in hemophilia A patients, are endocytosed by human MO-DCs through mannose sensitive pathway (14), bind to CD206 (18), and present mannose-exposed sugars at positions N239 and N2118 (17).

A 4389-base pair fragment was amplified by PCR from a cDNA encoding a partially BDD-FVIII (Δ2FVIII) (22) and introduced into the pcDNA3.1/V5-His-TOPO-TA vector (Thermo Fisher Scientific, Waltham, MA, United States). Δ2FVIII contains the 30 N-terminal amino-acids of the B domain of FVIII, and hence the N-glycosylation site NAT at position 757–759 (23). The pcDNA3.1-Δ2FVIII plasmid containing the Δ2FVIII cDNA was mutated using the QuickChange II XL mutagenesis kit (Stratagene, La Jolla, CA, United States). N239 and/or N2118 were mutated to Q, using the protocol provided by Stratagene. The wild-type and mutated Δ2FVIII cDNA were inserted into the pLIVE vector (Mirus, Madison, WI, United States).

### Cloning, Production and Purification of Wild-Type and Mutant B Domain-Deleted FVIII

cDNA encoding human BDD-FVIII (HSQ), containing the 14-amino acid segment SFSQNPPVLKRHQR in place of the B domain, cloned in the ReNeo plasmid (24) was used as a template to generate the FVIII^239Q^, FVIII^2118Q^, FVIII^2118A^, and FVIII^239Q/2118Q^ mutants by splicing-by-overlap extension mutagenesis. Presence of the mutations was confirmed by standard sequencing analysis. BHK-M cells (a kind gift from Prof P. Lollar, Emory University, Atlanta, Georgia, United States) were transfected and selected for neomycin resistant clones using Geneticin- sulfate (500 μg/ml, Sigma Aldrich, St-Louis, MO, United States). Screening of FVIII producing clones was performed by detection of the FVIII:antigen (FVIII:Ag) that refers to the amount of FVIII protein and FVIII:C that refers to the detectable pro-coagulant activity of FVIII. FVIII:Ag was detected by a sandwich ELISA using an anti-FVIII light chain specific monoclonal antibody (mAb) (Clone ESH-8, BioMedica Diagnostics, Stanford, CA, United States), and a biotinylated anti-FVIII heavy chain mAb (Clone GMA-8015, Green Mountain Antibodies, Burlington, United States), as capture and detection antibodies. FVIII:C was measured by chromogenic assay (Siemens Healthcare Diagnostic, Marburg, Germany). For quantification of FVIII in supernatants and cell lysates, a sandwich ELISA with ESH8 mAb and the biotinylated SAF8C polyclonal Ab, as capture and detection antibodies was performed. The stable expression of wild-type and mutated FVIII by BHK-M cells, and FVIII purification were performed as previously described (13). Briefly, the highest expressing clones for each FVIII was selected (according to the quantity of FVIII:C produced by 1.10^6^ million cells/ml in 24 h) and were scaled up to near confluency before switching the medium to serum-free AIM-V medium (Thermo Fisher Scientific). Medium was collected every 24 h and cells were replenished with fresh AIM-V medium. FVIII purification was performed by affinity chromatography on VIII select column (GE Healthcare, Chicago, IL, United States), followed by anion-exchange chromatography on HiTrap Resource Q column (GE Healthcare), as previously described (13). Purified FVIII was analyzed by 4–12% gradient SDS-PAGE (1 μg per well) with and without activation by bovine thrombin (Sigma-Aldrich). A silver staining of the gel was performed to detect proteins. Specific activity of purified WT and FVIII mutants were evaluated by chromogenic assay and absorbance at 280 nm with molar extinction coefficient of 256,300 M^–1^cm^–1^.

### Cloning and Site-Directed Mutagenesis of the Light Chain of FVIII

A N2118A mutated light chain of human FVIII was amplified using appropriate primers from the commercially available cDNA (pSP64-VIII, ATCC, Manassas, VA, United States). The mutated light chain fragment was cloned in the pNUT vector, with the signal sequence of human IgG kappa. The mutated light chain was produced in DMEM/F12, 5% fetal calf serum (FCS) by chinese hamster ovary (CHO) K1 stably transfected, and purified by ion-exchange chromatography. Briefly, a Resource S column column (GE Healthcare) was equilibrated with 10 mM histidine-HCl, 5 mM CaCl_2_, 250 mM NaCl, 0.01% tween 80 (pH 6.0). The culture supernatant in 10 mM MES, 5 mM EDTA, 0.01% tween 80, pH 6.0 was injected on the column. The recombinant light chain was eluted with a NaCl gradient, dialyzed in RPMI for 2 h at 4°C and quantified by sandwich ELISA using the human monoclonal anti-C2 conformational epitope-specific IgG (clone BO2C11, gift from JM Saint Rémy, Katholieke Universiteit Leuven, Leuven, Belgium), and ESH8 monoclonal antibody. Human recombinant FVIII was used as a standard. The wild-type light chain was a kind gift from Dr. E. L. Saenko (Center for Vascular and Inflammatory Diseases, University of Maryland School of Medicine, Baltimore, United States) and had been prepared from human FVIII as described (25).

### FVIII Gene Transfer to FVIII-Deficient Mice and Correction of Bleeding Time

**FIGURE 1:**
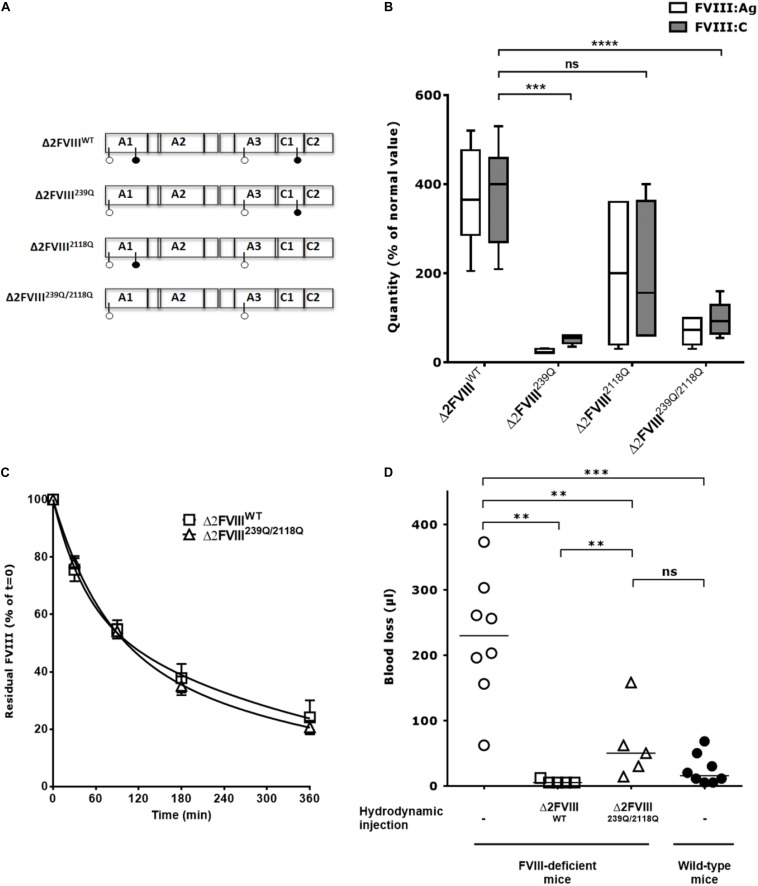
*In vivo* production of wild-type and mutated B domain-deleted FVIII lacking the N-glycosylation sites at 239 and 2118. **(A)** Human BDD-FVIII was modified by site-directed mutagenesis. The four glycosylation sites are depicted with pin symbols, the full pins represent N239 and N2118, on A1 and C1 domain, respectively. Four constructs were generated from the Δ2FVIII sequence: Δ2FVIII wild-type FVIII (Δ2FVIII^WT^), single N239Q mutant (Δ2FVIII^239Q^), single N2118Q mutant (Δ2FVIII^2118Q^) and double mutant FVIII (Δ2FVIII^239Q/2118Q^). **(B)** The different pLIVE constructs were injected to FVIII-deficient mice (4 to 11 mice per group) and blood was recovered after 4 days. The graph depicts as boxes and whiskers the levels of FVIII in plasma, as measured by ELISA (FVIII:Ag, empty bars) and by functional chromogenic assay (FVIII:C, full bars) using normal human plasma as a standard. Results are a pool of two independent experiments. **(C)** FVIII-deficient mice received hydrodynamic injections of pLIVE constructs encoding wild-type (six mice, empty squares) and double mutant FVIII (six mice, empty triangles) FVIII. Six days later, activated NHS-biotin was injected to each mouse, and residual biotinylated FVIII was measured at indicated time points. Data represent the percentage of residual biotinylated FVIII (mean ± SEM) quantified at *t* = 0 (15 min after biotin injection) which was a set at 100% for each mouse. For calculation of half-life, the best fit was obtained with non-linear regression with two exponential decay (Prism v5.0b, GraphPad Software, Inc.). **(D)** Four days after hydrodynamic injections of pLIVE constructs encoding wild-type (five mice, empty squares) and double mutants FVIII (five mice, empty triangles), FVIII-deficient mice were subjected to tail clip experiments and blood loss was measured. Untreated FVIII-deficient mice (8 mice, empty circles) and wild-type C57BL/6 mice (eight mice, full circles) were used as controls. Horizontal bars depict medians. Statistical analysis was performed using the non-parametric two-tailed Mann-Whitney *U* test with a confidence interval of 95%. **P < 0.01; ***P < 0.001; ****P < 0.0001; *ns:* not significant.

FVIII-deficient mice were injected in the tail vein with pLIVE plasmid (100 μg) encoding wild-type FVIII (Δ2FVIII) (22), or the N239Q (Δ2FVIII^239Q^), N2118Q (Δ2FVIII^2118Q^) or N239Q/N2118Q (Δ2FVIII^239Q/2118Q^) mutants (Figure 1A), in a large volume (10% of body weight) of 0.9% NaCl within 5 s (26). Four days later, blood was collected. FVIII:C and FVIII:Ag were quantified in citrated plasma using a chromogenic assay (Siemens Healthcare Diagnostics) and an Asserachrom kit (Stago, Asnières-sur-Seine, France), respectively. On day five, mice were anesthetized and 3 mm of the distal tail were cut, blood was collected during 30 min in 50 ml of saline buffer at 37°C. Erythrocytes were then pelleted at 1500 g and lysed in H_2_O. The amount of released hemoglobin, proportional to blood loss, was determined by measuring the optical density at 416 nm, using a standard curve prepared upon lysis of 20 to 100 μl of mouse blood. The half-life of endogenously produced FVIII was performed as previously described (27). Briefly, 6 days after the hydrodynamic injection, mice were injected intravenously with 500 μg of biotin-N-hydroxysuccinimide ester (Pierce, Rockford, United States) dissolved in saline buffer. Blood was collected on citrated tubes at different time points after injection and residual biotinylated FVIII was measured by ELISA, with a polyclonal anti-human FVIII antibody (SAF8C, Kordia, Leiden, Netherlands) as capture antibody and streptavidin-HRP for detection.

### FVIII Uptake by Human and Mouse DCs

Human DCs were prepared from purified monocytes (MO) by CD14 positive selection (Miltenyi Biotec, Paris, France), cultured in RPMI-1640 (Lonza, Verviers, Belgium), 10% FCS (Life Technologies, Saint-Aubin, France), supplemented with GM-CSF (1000 IU/10^6^ cells) and IL-4 (500 IU/10^6^ cells) (Miltenyi Biotec) (13). Buffy bags from anonymous healthy donors who gave informed consent were obtained from the Etablissement Français du Sang (EFS, Rungis, France), in accordance with EFS guidelines. After 5 days, MO-DC differentiation was validated by flow cytometry using CD1a and CD14 staining (BD Pharmingen, San Jose, CA, United States). Murine DCs were generated by isolation of bone marrow cells from SureL1 mice cultured for 10 days in RPMI-1640, containing 10% FCS, 50 mM 2-mercaptoethanol and 200 U/ml murine GM-CSF (Cellgenix Technology Transfer, Freiburg, Germany) (28). The purity of bone marrow derived-DCs (BM-DCs) was assessed by flow cytometry using CD11c staining. FVIII endocytosis by DCs was detected using the monoclonal anti-FVIII IgG, mAb 77IP52H7, conjugated to FITC after permeabilization of cells with 0.1% saponin (14).

### Activation of FVIII-Specific T Cells

As a source of FVIII-specific T-cells, the murine FVIII-specific T-cell hybridoma 1G8-A2, which was generated by immunizing SureL1 mice with BDD-FVIII (see Supplementary Material) (18), and the human FVIII-specific T-cell line D9E9 (from Dr. M. Jacquemin) (29) were used. Five day old MO-DCs from DRB1^∗^0101 healthy donors or mitomycin C-treated splenocytes from SureL1 mice were incubated for 24 h with the FVIII-specific T-cell hybridoma 1G8-A2 (10,000 MO-DCs or 200,000 splenocytes for 100,000 T cells) with FVIII in X-VIVO^15^ medium (Lonza). Levels of secreted interleukin-2 (IL-2) were assessed using BD OptEIA mouse IL-2 ELISA set (BD Biosciences). When the human FVIII-specific T-cell line D9E9 was used, 5,000 T cells were incubated with either MO-DCs from a DRB1^∗^1501 donor, or with the FVIII-specific human B-cell line (BO2C11) expressing the DRB1^∗^1501/DRB5^∗^0101 alleles (10,000 cells) (30) in DMEM-F12 media (Lonza) containing 10% FCS, 20 IU/ml human IL-2 (Sigma Aldrich), and FVIII fragments for 20 h at 37°C. When indicated, MO-DCs or BO2C11 were pre-incubated 30 min at 37°C with EDTA (5 mM) or mannan (1 mg/ml) prior to incubation with FVIII and T cells. The production of interferon-gamma was measured in the supernatants using the human IFN-gamma Duo Set (R&D Systems, Minneapolis, Minnesota, United States).

### Administration of FVIII and Characterization of the Anti-FVIII Immune Response

To evaluate the half-life of recombinant FVIII, FVIII-deficient mice were administered intravenously with FVIII^HSQ^ or FVIII^2118Q^ (100 μl, 10 nM). Plasma was collected at different time points and FVIII:Ag was measured by ELISA using the anti-light chain mAb ESH-8, and an anti-FVIII heavy chain mAb GMA-8015, as coating and detection antibodies, respectively, and human plasma as a standard (Siemens Healthcare Diagnostics). To investigate the immunogenicity of recombinant FVIII, FVIII-deficient mice or VWF/FVIII-deficient mice were injected intravenously with 0.5 μg FVIII^HSQ^ or FVIII^2118Q^, once a week for 4 weeks. Endotoxin levels in the different recombinant FVIII were below the accepted threshold (i.e., <0.01 ng endotoxin/20 g mouse weight) as assessed using the ToxinSensor Chromogenic LAL Endotoxin Assay Kit (Genscript, Piscataway, NJ, United States). Blood was collected 5 days after the last FVIII injection. ELISA plates were coated with FVIII (1 μg/ml, Recombinate, Baxter, Maurepas, France) overnight at 4°C. After blocking with PBS-1% BSA, plasma was incubated for 1 h at 37°C. Bound IgG were revealed with a HRP-coupled polyclonal goat anti-mouse IgG antibody (Southern Biotech, Anaheim, CA, United States) and the OPD substrate. The mouse monoclonal anti-FVIII IgG mAb6 (from Dr. J. M. Saint-Remy) was used as a standard. FVIII inhibitors were measured by incubating heat-inactivated mouse plasma with human standard plasma for 2 h at 37°C. FVIII residual pro-coagulant activity was measured by chromogenic assay. Results are expressed in Bethesda titers (BU/ml) that correspond to the reciprocal dilution of the mouse plasma that yields 50% residual FVIII activity.

## Results

### Elimination of the N239 and N2118 Glycosylation Sites Is Compatible With the Production and Pro-coagulant Function of FVIII

The cDNA of human recombinant BDD-FVIII (Δ2FVIII) (22, 23) was modified by site-directed mutagenesis to replace N239 and/or N2118 with Gln residues and cloned into the pLIVE vector for hydrodynamic injection experiments (Figure 1A). Mice injected with pLIVE encoding wild-type Δ2-FVIII (Δ2FVIII^WT^) produced 370 ± 37 IU/ml FVIII:C or 373 ± 35 IU/ml FVIII:Ag (mean ± SEM, Figure 1B). Mice injected with Δ2FVIII^239Q^, Δ2FVIII^2118Q^ or Δ2FVIII^239Q/2118Q^-encoding pLIVE produced 52 ± 5, 193 ± 82 and 97 ± 10 IU/ml FVIII:C, or 25 ± 2, 200 ± 72 and 69 ± 8 IU/ml FVIII:Ag, respectively, indicating a 2 to 14-fold reduction in circulation levels of FVIII in mice depending on the constructs.

The half-lives of the endogenously produced FVIII were evaluated following injection of activated NHS-biotin. FVIII clearance in FVIII-deficient mice followed a double-exponential distribution. Half-lives of Δ2FVIII^WT^ in the fast and slow phases of elimination were 30 and 260 min, respectively, with 38% of the FVIII being removed in the fast phase (*R*^2^ = 0.91, Figure 1C). Half-lives of Δ2FVIII^239Q/2118Q^ in the fast and slow phases of elimination were 48 and 276 min, respectively, with 50% of the FVIII being removed in the fast phase (*R*^2^ = 0.96). Thus, the half-lives of Δ2FVIII^WT^ and Δ2FVIII^239Q/2118Q^ were similar.

We further analyzed the capacity of endogenously produced Δ2FVIII^WT^ and Δ2FVIII^239Q/2118Q^ to correct blood loss in tail clipping experiments. We first confirmed that wild-type non-hemophilic mice lose significantly less blood than FVIII-deficient mice (*P* = 0.001, Figure 1D). The hydrodynamic injection of Δ2FVIII^WT^ or Δ2FVIII^239Q/2118Q^-encoding plasmids into FVIII-deficient mice corrected blood loss as compared to untreated mice (*P* = 0.004 and *P* = 0.013, respectively). Interestingly, blood loss in FVIII-deficient mice treated with the Δ2FVIII^239Q/2118Q^ construct was not significantly different from that of wild-type non-hemophilic mice. The fact that FVIII-deficient mice treated with the Δ2FVIII^WT^-encoding plasmid bled significantly less than mice treated with the Δ2FVIII^239Q/2118Q^-encoding plasmid (*P* = 0.010) is probably due to the fact that circulating levels of FVIII were significantly greater in the former group of mice (373 ± 35 IU/ml, mean ± SEM, Figure 1B) than in Δ2FVIII^239Q/2118Q^-treated mice (69 ± 8 IU/ml).

### Production and Characterization of Recombinant FVIII Mutants Lacking the N239 and N2118 Glycosylation Sites

**FIGURE 2:**
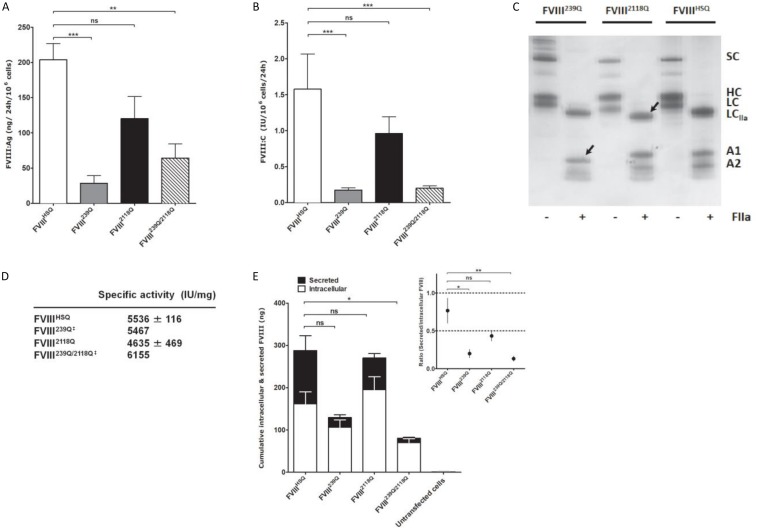
Production of recombinant wild-type and of mutated B domain-deleted FVIII lacking the N-glycosylation sites at 239 and/or 2118. Levels of FVIII:Ag **(A)** and FVIII:C **(B)** produced from BHK-M cells transfected with either the FVIII^HSQ^, FVIII^239Q^, FVIII^2118Q^, or FVIII^239Q/2118Q^ transgenes were measured by ELISA and functional chromogenic assay, respectively, and are expressed relative to 1.10^6^ cells. Results are representatives of two independent experiments (mean ± SEM), *n* = 7–10 depending on the construct. Statistical comparisons between cells transfected with mutants and FVIII^HSQ^ were made using the two-tailed Mann-Whitney *U* test. **(C)** SDS-PAGE of FVIII^HSQ^, FVIII^239Q^, and FVIII^2118Q^ with and without exposure to thrombin (FIIa). SC, single chain FVIII; HC, FVIII heavy chain (A1-A2 domains); LC, FVIII light chain; LC_IIa_, thrombin-cleaved light chain; A1, A1 domain; A2, A2 domain. Arrow heads depict the deglycosylated A1 domain and light chain for FVIII^239Q^ and FVIII^2118Q^, respectively. **(D)** FVIII activity was measured by functional chromogenic assay and protein concentration was evaluated by absorbance at 280 nm. ^‡^FVIII^239Q^ and FVIII^239Q/2118Q^ were purified and tested only once. **(E)** Levels of secreted and intracellular FVIII produced by transfected and untransfected BHK-M cells were measured after 24 h of culture. Statistical analysis was performed on intracellular levels of FVIII using two-way ANOVA test. The inset depicts the ratios of secreted versus intracellular FVIII levels. Statistical comparison was assessed by a *t*-test. Data represent means ± SEM of three independent experiments. *P < 0.05, **P < 0.01; ***P < 0.001; *ns:* not significant.

We then assessed the production of wild-type BDD-FVIII (FVIII^HSQ^) and of the FVIII^239Q^, FVIII^2118Q^, and FVIII^239Q/2118Q^ mutants by stably transfected BHK-M cell clones. Rates of production of FVIII^2118Q^ (FVIII:C = 0.96 ± 0.23 IU/10^6^cells/24 h; FVIII:Ag = 120 ± 31 ng/10^6^cells/24 h; mean ± SEM, Figures 2A,B, respectively) were not statistically different from that of FVIII^HSQ^ (FVIII:C = 1.58 ± 0.49 IU/10^6^cells/24 h; FVIII:Ag = 204 ± 23 ng/10^6^cells/24 h). In contrast, FVIII^239Q^ (0.17 ± 0.03 IU/10^6^cells/24 h; 29 ± 11 ng/10^6^cells/24 h) and FVIII^239Q/2118Q^ (0.20 ± 0.03 IU/10^6^cells/24 h; 64 ± 20 ng/10^6^cells/24 h) were produced at rates 8 to 9-fold lower than FVIII^HSQ^. The absence of N-linked glycans at positions 239 and 2118 was validated by comparing the migration profiles of thrombin-digested purified FVIII^HSQ^ and mutants on SDS-PAGE. The lower molecular weight of the A1 domain of the thrombin-digested FVIII^239Q^ mutant and of the light chain of the thrombin-digested FVIII^2118Q^ mutant, as compared to the A1 domain and light chain of the thrombin-digested FVIII^HSQ^ confirmed the removal of the N-linked glycosylation sites (Figure 2C). Besides, the similar molecular weights observed for the A1 fragment (digested FVIII) in the case of FVIII^HSQ^ and FVIII^2118Q^ and the digested light chain of the FVIII^239Q^ and FVIII^HSQ^ suggests that site directed mutagenesis at N239 or/and N2118 did not affect the N-glycosylations at positions N41 and N1810, respectively. MS analysis would confirm this observation. Further, a digestion of FVIII^HSQ^ and FVIII^2118Q^ with the N-glycosidase F was performed to confirm that the lower molecular weight of the light chain is due to the removal of N-linked glycosylation at N2118 and not due to an uncontrolled proteolytic cleavage (Supplementary Figure S1). The purified FVIII mutants exhibited specific activities between 4600–6200 IU/mg (Figure 2D) similar to that of FVIII^HSQ^. Furthermore, FVIII^HSQ^ and the FVIII mutants bound similarly to immobilized VWF and phosphatidylserine (Supplementary Figure S2). In order to decipher whether the removal of N239 or/and N2118 affects the production and/or secretion of FVIII, we measured secreted (supernatant) and intracellular (cell lysate) FVIII after 24 h of culture of BHK-M cells. Intracellular FVIII levels were similar for the FVIII^239Q^, FVIII^2118Q^ and FVIII^HSQ^ transfected cells (106 ± 18 ng/10^6^ cells; 195 ± 31 ng/10^6^ cells; 161 ± 28 ng/10^6^ cells, respectively). In contrast, the intracellular FVIII levels were significantly decreased in the case of FVIII^239/2118Q^ (70 ± 9 ng/10^6^ cells/24 h, *P* < 0.05, Figure 2E). Comparison of the ratios of secreted versus intracellular FVIII levels (Figure 2E, inset) confirms that the N239Q mutation, either alone or in combination with the N2118Q mutation, is associated with a hampered secretion of FVIII.

### Removal of the N2118 Glycosylation Abrogates FVIII Presentation by Human Dendritic Cells

**FIGURE 3:**
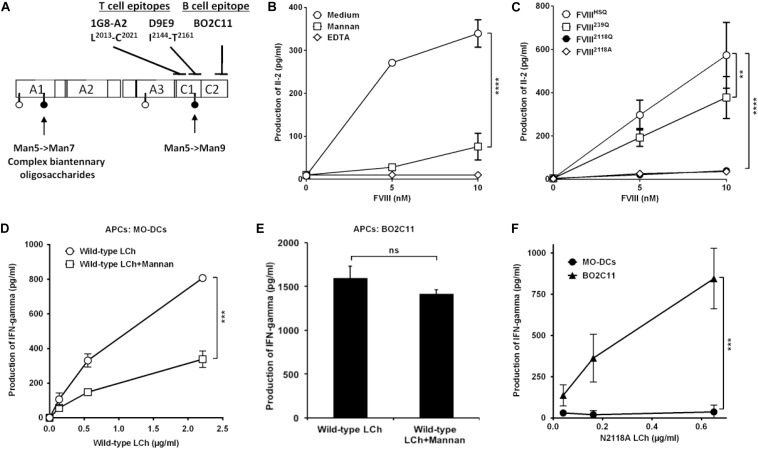
Removal of N-glycosylation at 2118 site alters FVIII light chain presentation by human dendritic cells. **(A)** Sites of mutations of FVIII and epitope specificity of FVIII specific T and B cells used in our assays. The figure depicts the structure of human FVIII and the determined sites of N-glycosylation as reported (15), represented as pins. The nature of oligosaccharides expressed at N239 and N2118 is emphasized: while N2118 carries only Man5- >Man9 oligomannose carbohydrates, N239 bears an heterogenous population of oligomannose- and complex-type carbohydrates. The epitopes for 1G8-A2, a FVIII-specific mouse CD4 + T-cell hybridoma (epitope L^2012^-C^2021^), D9E9, a human FVIII-specific T-cell line (epitope: I^2144^-T^2161^), and for BO2C11, a human FVIII-specific B-cell line (epitope: 2170–2332) (29, 30) are also depicted. **(B)** HLA-matched human MO-DCs were pre-incubated with mannan or EDTA prior to incubation with FVIII and 1G8-A2. **(C)** Activation of 1G8-A2 by FVIII-loaded HLA-matched human MO-DCs with FVIII^HSQ^, FVIII^239Q^, FVIII^2118Q^, or FVIII^2118A^ was evaluated by quantification of secreted Il-2 in the supernatant by ELISA. Representative of three experiments (mean ± SD), *n* = 3. **(D–F)** Presentation of wild-type or mutant FVIII light chain to D9E9. Five-day-old MO-DCs **(D)** or BO2C11 B cells **(E)** were pre-incubated in medium alone or in the presence of mannan prior to incubation with D9E9 and wild-type FVIII light chain (LCh). MO-DCs or BO2C11 B cells **(F)** were incubated with increasing concentrations of mutated N2118A light chain (LCh) in the presence of D9E9. D9E9 activation was assessed by measuring IFN-gamma in the supernatant by ELISA (mean ± SD), *n* = 3. Representative of two independent experiments. Statistical differences were assessed using two-way ANOVA test. **P < 0.01; ***P < 0.001; ****P < 0.0001; *ns:* not significant.

To investigate the potential of the purified FVIII^HSQ^ and mutants to be presented to CD4+ T cells by human APCs, we generated a mouse T-cell hybridoma specific for human FVIII and restricted to the HLA-DRB1^∗^01:01 allele, referred to as 1G8-A2. The activation of 1G8-A2 by FVIII-loaded human MO-DCs from a HLA-DRB1^∗^01:01 donor was evaluated by measuring IL-2 secretion. 1G8-A2 is specific for the ^2013^LFLVYSNKC^2022^ core peptide, located in the C1 domain of FVIII light chain (Figure 3A and Supplementary Figure S3). As previously reported (10), T-cell activation was drastically reduced when MO-DCs were pre-incubated with mannan (1 mg/ml) or EDTA (5 mM) prior to incubation with FVIII^HSQ^ (Figure 3B). Likewise, removal of the N2118 glycosylation site resulted in a drastic reduction in 1G8-A2 activation by MO-DCs (39.5 ± 13.4 pg/ml, mean ± SD as compared to the use of FVIII^HSQ^ (572.9 ± 151.9 pg/ml, *P* < 0.0001 at 10 nM FVIII, Figure 3C). A different FVIII variant with an N2118A instead of an N2118Q mutation also failed to activate 1G8-A2 (35.3 ± 2.1 pg/ml, *P* < 0.001 as compared to FVIII^HSQ^). In contrast, co-incubation of cells with the FVIII^239Q^ mutant yielded a significant though less marked reduction in IL-2 secretion (377.7 ± 96.8 pg/ml, *P* = 0.002 as compared to FVIII^HSQ^). Of note, removal of both N239 and N2118 glycosylation sites did not have an additional effect on T-cell activation over the removal of the N2118 site alone (Supplementary Figure S4).

To confirm this observation, we used a different experimental set-up (different HLA context and using a T-cell line with a different epitope specificity), in which we compared the presentation of a N2118A mutated or wild-type FVIII light chain (25) to the HLA-DRB1^∗^15:01-restricted human D9E9 T-cell line which is specific for the ^2144^IIARYIRLHPTHYSIRST^2164^ C1 domain peptide (29) (Figure 3A). As a source of APCs, we compared HLA-matched MO-DCs to the human BO2C11 B-cell line (30). The activation of D9E9 T cells by MO-DCs incubated with the wild-type FVIII light chain was drastically reduced in the presence of mannan (Figure 3D), thus reproducing the data obtained with complete FVIII^HSQ^ (Figure 3B). In contrast, BO2C11 B cells activated D9E9 T cells when incubated in the presence of either wild-type or mutated light chains (Figures 3E,F), indicating that the absence of the mannose-ending glycan on the C1 domain does not alter the conformation of the BO2C11 target epitope on the FVIII C2 domain. It also confirmed that FVIII light chain endocytosis by BO2C11 B cells, which is mediated by the B-cell receptor, is insensitive to the presence of mannan (Figure 3E). Since removal of the glycans at position N2118 was sufficient to significantly reduce FVIII presentation to CD4+ T cells, we restricted the rest of the study to FVIII^2118Q^.

### Mannose-Ending Glycans at Position 2118 do Not Modulate FVIII Endocytosis and Presentation by Mouse APCs

**FIGURE 4:**
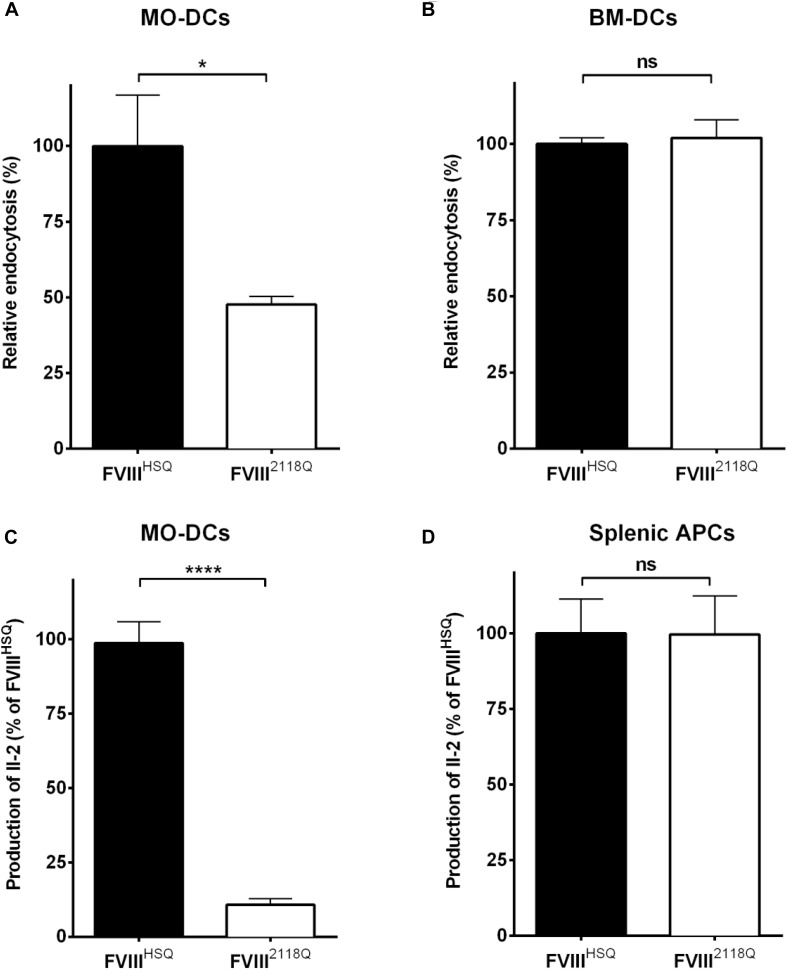
Removal of N-glycosylation at 2118 does not alter FVIII presentation by mouse APCs. **(A,B)** Immature human MO-DCs **(A)** or mouse BM-DCs **(B)** were incubated with FVIII^HSQ^ or FVIII^2118Q^ (20 nM) for 1 h. Internalized FVIII was detected by fluorescence-activated cell sorting (FACS) with LSR II and FACSDiva software. Results are expressed as the percentage of median fluorescence intensity (MFI), wherein 100% corresponds to the MFI obtained with wild-type FVIII (FVIII^HSQ^). **(C,D)** The activation of 1G8-A2 by FVIII-loaded (10 nM) HLA-matched human MO-DCs **(C)** or splenocytes from SURE-L1 mice **(D)** was evaluated. IL-2 produced by the activated T cells was measured in the supernatant after 24 h. Mean ± SD of three independent experiments. Statistical analysis was performed using two-tailed, unpaired *t*-test. *P < 0.05; ****P < 0.0001; *ns:* not significant.

We have shown earlier that mouse BM-DCs, in contrast to human MO-DCs, do not endocytose FVIII through mannose-sensitive endocytosis pathways (31). Here, we compared the ability of human MO-DCs and HLA-DRB1^∗^01:01-transgenic Sure-L1 mouse DCs to endocytose and present FVIII^HSQ^ and FVIII^2118Q^ to the FVIII-specific T-cell hybridoma 1G8-A2. While a 50% reduction of endocytosis by human MO-DCs was observed with FVIII^2118Q^ compared to FVIII^HSQ^ (Figure 4A), mouse BM-DCs internalized FVIII^HSQ^, and FVIII^2118Q^ in a similar manner (Figure 4B). Likewise, mouse splenic APCs activated 1G8-A2 in a similar manner irrespective of the source of FVIII used: FVIII^HSQ^ or FVIII^2118Q^ (Figure 4D) whereas only around 10% of T-cell activation by human MO-DCs was measured with FVIII^2118Q^ (Figure 4C).

### The Absence of N2118 Mannose-Ending Glycan Does Not Reduce FVIII Immunogenicity in Mice

**FIGURE 5:**
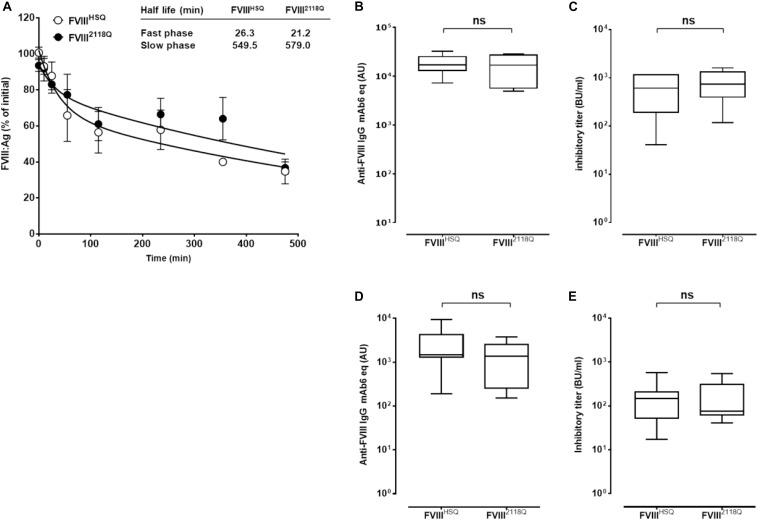
Removal of 2118 mannose ending glycans does not modulate FVIII immunogenicity in FVIII-deficient mice, neither in double VWF/FVIII-deficient mice. **(A)** Half-life of wild type and N2118Q mutated FVIII. FVIII^HSQ^ and FVIII^2118Q^ were administered to FVIII-deficient mice and the residual FVIII:Ag was measured at different time points (*n* = 6 mice per time point) using a sandwich ELISA. The data is plotted as percentage of initial FVIII activity (measured 5 min after administration) versus time (mean ± SEM). Representative of two independent experiments. Inset. The slow and fast phases of FVIII clearance were determined by fitting the data to a two phase decay curve. The immunogenicity of FVIII^2118Q^ was evaluated in FVIII-deficient mice: **(B,C)**, seven mice for both FVIII^HSQ^ and FVIII^2118Q^ group and VWF/FVIII double-deficient mice; **(D,E)** 8 and 6 mice for FVIII^HSQ^ and FVIII^2118Q^ group, respectively. One week after the fourth injection, blood samples were collected and anti-FVIII IgG titers **(B,D)** quantified using a purified mouse monoclonal anti-FVIII IgG (Mab6) as a standard. Results are expressed in arbitrary units (AU). Inhibitory titers toward FVIII **(C,E)** were measured by chromogenic assay. Statistical analysis was performed using the non-parametric two-tailed Mann-Whitney *U* test. *ns*, not significant.

We then investigated the consequence of removing the N2118 glycosylation site on FVIII immunogenicity in FVIII-deficient mice. First, we confirmed that kinetics of FVIII elimination in both the slow and fast phases did not differ statistically between FVIII^HSQ^ and FVIII^2118Q^ (Figure 5A), confirming the observations obtained with the endogenous expression of Δ2FVIII^239Q/2118Q^ (Figure 1C).

FVIII-deficient mice were injected with 10 nM FVIII^2118Q^ or FVIII^HSQ^ once a week for 4 weeks, and anti-FVIII IgG and inhibitory titers were measured in serum. The anti-FVIII IgG titers were similar whether mice were treated with FVIII^2118Q^ or FVIII^HSQ^ (17362 ± 9632 AU versus 18758 ± 3166 AU, respectively, mean ± SEM, Figure 5B). Likewise, the inhibitory titers in the serum of FVIII^2118Q^-treated mice were similar to that in the serum of FVIII^HSQ^-treated mice (810 ± 527 BU/ml versus 639 ± 455 BU/ml, respectively, Figure 5C). Next, we investigated the immunogenicity of FVIII^2118Q^ and FVIII^HSQ^ in the absence of VWF in double FVIII/VWF-deficient mice. As seen in the case of FVIII-deficient mice, neither anti-FVIII IgG titers nor inhibitory titers differed between double deficient mice treated with FVIII^HSQ^ (2712 ± 2599 AU; 174 ± 195 BU/ml, Figure 5D) or FVIII^2118Q^ (3979 ± 4384 AU; 180 ± 175 BU/ml, Figure 5E). Taken together, our results indicate that oligomannose carbohydrates at N2118 do not play a major role in FVIII immunogenicity, at least in mice.

## Discussion

Missense mutations in 580 (42%) of the 1384 amino acids that encompass activated FVIII lead to moderate, mild or severe hemophilia A (HAMSTeRS database November 2016)^1^. This illustrates the critical relationship between the structure and function of proteins in general, and of FVIII in particular, which prompted us to estimate the repercussion of removing N-glycosylation sites of FVIII on its pro-coagulant activity. The recombinant FVIII^239Q/2118Q^ demonstrated specific activity similar to that of its respective non-mutated counterpart. Accordingly, Δ2FVIII^239Q/2118Q^ protected mice from experimentally induced blood loss, and FVIII^239Q/2118Q^ bound normally to VWF and phosphatidylserine. These observations indicate that removal of oligomannose carbohydrates at N239 and N2118 does not alter the pro-coagulant activity of FVIII.

Circulating levels of Δ2FVIII *in vivo* and levels of production of FVIII^HSQ^ *in vitro* were similar or 2-fold lower when the molecules were mutated at N2118 and 9 to 14-fold lower when mutations were located at N239. In support of our data, Wei *et al.* have shown that, in contrast to mutation at N2118, mutation at N239 drastically reduces FVIII production (32). At the cellular level, the efficient transport of FVIII from the endoplasmic reticulum (ER) to the Golgi apparatus requires the interaction of FVIII with the mannose-binding lectin LMAN1 (33). The interaction of FVIII with the carbohydrate recognition domain of LMAN1 implicates either high mannose-containing N-linked glycosylations, or protein-protein interactions (34). Our observation of a low ratio of secreted versus intracellular FVIII in the case of FVIII^239Q^ and FVIII^239/2118Q^ suggests an essential role for the N239 glycan in FVIII export. Comparatively, the N2118 glycan plays a negligible role in FVIII production and secretion. We hypothesize that removal of the N239 glycosylation site, which is highly conserved among orthologous FVIII molecules (32), synergizes with the absence of N-glycosylation sites of the B-domain that is missing in FVIII^HSQ^ and Δ2FVIII, resulting in inefficient intracellular transport of FVIII. Of note, the *in vivo* half-life of Δ2FVIII was not affected by the removal of the 239 and 2118 N-glycosylation sites. Accordingly, the half-lives of recombinant FVIII^HSQ^ and FVIII^2118Q^ were also similar. Taken together, our data suggest that the oligomannose carbohydrates at N239 and N2118 of FVIII, that mediate binding to CD206 expressed on human macrophages and dendritic cells (10, 18), and to CLEC4M expressed on human liver and lymphatic sinusoidal endothelial cells (35), or to the soluble lectin Galectin-1 (36) are not involved in FVIII catabolism, at least in mice.

The first step of the primary immune response to therapeutic FVIII is its uptake by APCs. Our earlier work had documented the importance of mannose-ending glycans on FVIII for its recognition and internalization by MO-DCs, used as a model of APCs, and its subsequent presentation to CD4+ T cells (10). The phenomenon was true for both full-length and BDD-FVIII, thus highlighting the contribution of mannose-ending glycans outside the B domain for FVIII recognition by human APCs (10, 14). In humans, the presence of high mannose glycans is not frequent on secreted proteins (37). The deliberate mannosylation of non-self-antigens has been shown to enhance their endocytosis by human DCs and subsequent presentation to antigen-specific T lymphocytes (38, 39). Indeed, human DCs express several endocytic C-type lectin receptors, including CD206, DC-SIGN and dectin 2, which bind exposed mannose residues on glycoproteins through their carbohydrate recognition domains (40, 41). Interestingly, CD206 is not only expressed by *in vitro* derived DCs, but also by some subsets of DCs in the skin, tonsils and blood (42). The present work demonstrates the prevalent role of mannosylated glycans at N2118 in mediating FVIII uptake by MO-DCs, in the context of the absence of the heavily glycosylated B domain. Mutations of N239 had only a marginal effect on T-cell activation by MO-DCs, which may relate to the fact that glycans at N239 contain high-mannose, as well as hybrid and complex structures, while glycans at N2118 exclusively contain high-mannose structures (Man 5-9) (15–17).

We and others have recently documented the crucial role played by charged residues in the C1 and C2 domains of FVIII for its recognition and endocytosis by MO-DCs (11, 13). Mutation to alanine residues of R2215 and R2220 in the protruding loops of the C2 domain of FVIII^HSQ^ (referred to as FVIII^R2215–20A^) as well as of R2090, K2092 and F2093 in the protruding loops of C1 (referred to as FVIII^C1^) independently reduced FVIII endocytosis by ≥50% and presentation to T cells by ≥75%. Importantly, FVIII^R2215–20A^ and FVIII^C1^ contain the N-linked high-mannose carbohydrates at position N2118. Conversely, FVIII^2118Q^ contains the native amino acids at positions 2092, 2093, 2090, 2215 and 2220, but is not presented to T cells by MO-DCs. These results point toward a critical role played by the C domains, and particularly the C1 domain, in the recognition and internalization of FVIII by MO-DCs. They also suggest that the engagement of the three FVIII entities – charged residues in the C1 domain, charged residues in the C2 domain and glycans at N2118, is required for FVIII uptake, and pave the way toward the identification of the surface receptors involved in the process.

Removal of the N-linked glycosylation site at position 2118 did not reduce the immunogenicity of FVIII in FVIII-deficient mice. This is reminiscent of the lack of reduced immunogenicity of the FVIII^R2215–20A^ and FVIII^C1^ in hemophilic mice, despite a drastic reduction in endocytosis by DCs (13). Interestingly, the immunogenicity of FVIII^C1^ was reduced as compared to that of FVIII^HSQ^ in double FVIII/VWF-deficient mice, thus highlighting a role for VWF in impairing the potential beneficial effect of mutations in C1 on a reduction of FVIII immunogenicity *in vivo.* We had previously identified CD206 as one of the receptors implicated in the mannose-sensitive uptake of FVIII by MO-DCs. The activation of T cells was partially blocked when MO-DCs and FVIII were incubated in the presence of an anti-CD206 antibody, and FVIII was demonstrated to interact with soluble and immobilized CD206 (10). Importantly, the interaction between FVIII and CD206 was inhibited in a dose-dependent manner by VWF (43). A role for endogenous VWF in preventing the reduction of FVIII^2118Q^ immunogenicity in FVIII-deficient mice is, however, not probable owing to the fact that there was no reduction in FVIII^2118Q^ immunogenicity in double FVIII/VWF-deficient mice.

The mutation of charged residues in the C1 and C2 domains inhibits endocytosis both by human MO-DCs and by mouse BM-DCs (12, 13). In contrast to human MO-DCs, however, mouse BM-DCs do not internalize FVIII through mannose-sensitive pathways (31). Accordingly, FVIII^2118Q^ and FVIII^HSQ^ were captured and presented to a similar extent to T cells when mouse BM-DCs or splenic APCs were used. Of note, we observed, as reported previously, that CD206 is expressed by mouse macrophages from the red pulp of the spleen (44), and by a subset of mouse splenic dendritic cells (45) that are present in freshly isolated splenocytes from Sure-L1 mice (data not shown), while in human spleen CD206 has been reported to be expressed by lining venous sinus cells and not by macrophages (46, 47). Overall, the organ-specific expression of C-type lectin receptors is different between mice and human (48). This is of importance in view of the fact that we have previously shown the accumulation of exogenously administered FVIII at the level of metallophilic macrophages in the marginal zone of the spleen of FVIII-deficient mice (49). The identification of preclinical animal models that better mimic the distribution of C-type lectin receptors in the human is necessary to confirm the reduced immunogenicity of FVIII^2118Q^ *in vivo*, and its potential safety for replacement therapy in patients with hemophilia A.

## Data Availability Statement

The datasets generated for this study are available on request to the corresponding author.

## Ethics Statement

Ethical review and approval was not required for the study on human participants in accordance with the local legislation and institutional requirements. Written informed consent for participation was not required for this study in accordance with the national legislation and the institutional requirements. The animal study was reviewed and approved by the Charles Darwin, Sorbonne Université, #2058.04.

## Author Contributions

SDe, SDa, CD, OC, JB, and SL-D designed the research. SDe, JR, SDa, BG, and OC performed the research. SDe, JR, SK, and SL-D analyzed the data. SDe, JB, and SL-D wrote the manuscript.

## Conflict of Interest

SDa, SK, and SL-D are inventors on a patent related to the research.

The remaining authors declare that the research was conducted in the absence of any commercial or financial relationships that could be construed as a potential conflict of interest.

## Footnotes

1 http://www.factorviii-db.org/

## References

[1] Ehrenforth S Kreuz W Scharrer I Kornhuber B. Factor VIII inhibitors in haemophiliacs. *Lancet.* (1992) 340:253 10.1016/0140-6736(92)90530-g 1353186

[2] Pavlova A Delev D Lacroix-Desmazes S Schwaab R Mende M Fimmers R Impact of polymorphisms of the major histocompatibility complex class II, interleukin-10, tumor necrosis factor-alpha and cytotoxic T-lymphocyte antigen-4 genes on inhibitor development in severe hemophilia A. *J Thromb Haemost.* (2009) 7:2006–15. 10.1111/j.1538-7836.2009.03636.x 19817985

[3] Astermark J Donfield SM Gomperts ED Schwarz J Menius ED Pavlova A The polygenic nature of inhibitors in hemophilia A: results from the Hemophilia Inhibitor Genetics Study (HIGS) Combined Cohort. *Blood.* (2013) 121:1446–54. 10.1182/blood-2012-06-434803 23223434 PMC3578958

[4] Dimitrov JD Dasgupta S Navarrete A-M Delignat S Repesse Y Meslier Y Induction of heme oxygenase-1 in factor VIII-deficient mice reduces the immune response to therapeutic factor VIII. *Blood.* (2010) 115:2682–5. 10.1182/blood-2009-04-216408 19890094

[5] James EA van Haren SD Ettinger RA Fijnvandraat K Liberman JA Kwok WW T-cell responses in two unrelated hemophilia A inhibitor subjects include an epitope at the factor VIII R593C missense site. *J Thromb Haemost.* (2011) 9:689–99. 10.1111/j.1538-7836.2011.04202.x 21251204 PMC4323178

[6] Matino D Gargaro M Santagostino E Di Minno MND Castaman G Morfini M IDO1 suppresses inhibitor development in hemophilia A treated with factor VIII. *J Clin Invest.* (2015) 125:3766–81. 10.1172/JCI81859 26426076 PMC4607121

[7] Varthaman A Lacroix-Desmazes S. Pathogenic immune response to therapeutic factor VIII: exacerbated response or failed induction of tolerance? *Haematologica.* (2019) 104:236–44. 10.3324/haematol.2018.206383 30514798 PMC6355482

[8] Lacroix-Desmazes S Navarrete A-M André S Bayry J Kaveri SV Dasgupta S. Dynamics of factor VIII interactions determine its immunologic fate in hemophilia A. *Blood.* (2008) 112:240–9. 10.1182/blood-2008-02-124941 18469198

[9] van Haren SD Herczenik E ten Brinke A Mertens K Voorberg J Meijer AB. HLA-DR-presented peptide repertoires derived from human monocyte-derived dendritic cells pulsed with blood coagulation factor VIII. *Mol Cell Proteomics.* (2011) 10:M110.002246. 10.1074/mcp.M110.002246 21467215 PMC3108829

[10] Dasgupta S Navarrete A-M Bayry J Delignat S Wootla B André S A role for exposed mannosylations in presentation of human therapeutic self-proteins to CD4+ T lymphocytes. *Proc Natl Acad Sci USA.* (2007) 104:8965–70. 10.1073/pnas.0702120104 17502612 PMC1885611

[11] Herczenik E van Haren SD Wroblewska A Kaijen P van den Biggelaar M Meijer AB Uptake of blood coagulation factor VIII by dendritic cells is mediated via its C1 domain. *J Allergy Clin Immunol.* (2012) 129:501–9, 509.e1–5. 10.1016/j.jaci.2011.08.029 21962992

[12] Wroblewska A van Haren SD Herczenik E Kaijen P Ruminska A Jin S-Y Modification of an exposed loop in the C1 domain reduces immune responses to factor VIII in hemophilia A mice. *Blood.* (2012) 119:5294–300. 10.1182/blood-2011-11-391680 22498747 PMC3680040

[13] Gangadharan B Ing M Delignat S Peyron I Teyssandier M Kaveri SV The C1 and C2 domains of blood coagulation factor VIII mediate its endocytosis by dendritic cells. *Haematologica.* (2016) 102:271–81. 10.3324/haematol.2016.148502 27758819 PMC5286935

[14] Repessé Y Dasgupta S Navarrete A-M Delignat S Kaveri SV Lacroix-Desmazes S. Mannose-sensitive receptors mediate the uptake of factor VIII therapeutics by human dendritic cells. *J Allergy Clin Immunol.* (2012) 129:1172–3; author reply 1174–5. 10.1016/j.jaci.2012.01.048<pmid< 22336079

[15] Medzihradszky KF Besman MJ Burlingame AL. Structural characterization of site-specific N-glycosylation of recombinant human factor VIII by reversed-phase high-performance liquid chromatography-electrospray ionization mass spectrometry. *Anal Chem.* (1997) 69:3986–94. 9322435 10.1021/ac970372z

[16] Kannicht C Ramström M Kohla G Tiemeyer M Casademunt E Walter O Characterisation of the post-translational modifications of a novel, human cell line-derived recombinant human factor VIII. *Thromb Res.* (2013) 131:78–88. 10.1016/j.thromres.2012.09.011 23058466

[17] Canis K Anzengruber J Garenaux E Feichtinger M Benamara K Scheiflinger F In-depth comparison of N-glycosylation of human plasma-derived factor VIII and different recombinant products: from structure to clinical implications. *J Thromb Haemost.* (2018) 16:1592–603. 10.1111/jth.14204 29888865

[18] Delignat S Repessé Y Gilardin L Dimitrov JD Lone YC Kaveri SV Predictive immunogenicity of Refacto AF. *Haemophilia.* (2014) 20:486–92. 10.1111/hae.12348 24372710

[19] Lee SJ Evers S Roeder D Parlow AF Risteli J Risteli L Mannose receptor-mediated regulation of serum glycoprotein homeostasis. *Science.* (2002) 295:1898–901. 10.1126/science.1069540 11884756

[20] Bi L Lawler AM Antonarakis SE High KA Gearhart JD Kazazian HH. Targeted disruption of the mouse factor VIII gene produces a model of haemophilia A. *Nat Genet.* (1995) 10:119–21. 10.1038/ng0595-119 7647782

[21] Pajot A Michel M-L Fazilleau N Pancré V Auriault C Ojcius DM A mouse model of human adaptive immune functions: HLA-A2.1-/HLA-DR1-transgenic H-2 class I-/class II-knockout mice. *Eur J Immunol.* (2004) 34:3060–9. 10.1002/eji.200425463 15468058

[22] Meulien P Faure T Mischler F Harrer H Ulrich P Bouderbala B A new recombinant procoagulant protein derived from the cDNA encoding human factor VIII. *Protein Eng.* (1988) 2:301–6. 3150544 10.1093/protein/2.4.301

[23] Bihoreau N Paolantonacci P Bardelle C Fontaine-Aupart MP Krishnan S Yon J Structural and functional characterization of Factor VIII-delta II, a new recombinant Factor VIII lacking most of the B-domain. *Biochem J.* (1991) 277(Pt 1):23–31. 10.1042/bj2770023 1906711 PMC1151186

[24] Healey JF Barrow RT Tamim HM Lubin IM Shima M Scandella D Residues Glu2181-Val2243 contain a major determinant of the inhibitory epitope in the C2 domain of human factor VIII. *Blood.* (1998) 92:3701–9. 9808564

[25] Saenko EL Shima M Gilbert GE Scandella D. Slowed release of thrombin-cleaved factor VIII from von Willebrand factor by a monoclonal and a human antibody is a novel mechanism for factor VIII inhibition. *J Biol Chem.* (1996) 271:27424–31. 8910322 10.1074/jbc.271.44.27424

[26] Marx I Lenting PJ Adler T Pendu R Christophe OD Denis CV. Correction of bleeding symptoms in von Willebrand factor-deficient mice by liver-expressed von Willebrand factor mutants. *Arterioscler Thromb Vasc Biol.* (2008) 28:419–24. 10.1161/ATVBAHA.107.159442 18187670

[27] Badirou I Kurdi M Rayes J Legendre P Christophe OD Lenting PJ von Willebrand factor clearance does not involve proteolysis by ADAMTS-13. *J Thromb Haemost.* (2010) 8:2338–40. 10.1111/j.1538-7836.2010.04012.x 20704649

[28] Lutz MB Kukutsch N Ogilvie AL Rössner S Koch F Romani N An advanced culture method for generating large quantities of highly pure dendritic cells from mouse bone marrow. *J Immunol Methods.* (1999) 223:77–92. 10.1016/s0022-1759(98)00204-x 10037236

[29] Jacquemin M Vantomme V Buhot C Lavend’homme R Burny W Demotte N CD4+ T-cell clones specific for wild-type factor VIII: a molecular mechanism responsible for a higher incidence of inhibitor formation in mild/moderate hemophilia A. *Blood.* (2003) 101:1351–8. 10.1182/blood-2002-05-1369 12393451

[30] Jacquemin MG Desqueper BG Benhida A Vander Elst L Hoylaerts MF Bakkus M Mechanism and kinetics of factor VIII inactivation: study with an IgG4 monoclonal antibody derived from a hemophilia A patient with inhibitor. *Blood.* (1998) 92:496–506. 10.1182/blood.v92.2.496.414k16_496_506 9657749

[31] Delignat S Repessé Y Navarrete A-M Meslier Y Gupta N Christophe OD Immunoprotective effect of von Willebrand factor towards therapeutic factor VIII in experimental haemophilia A. *Haemophilia.* (2012) 18:248–54. 10.1111/j.1365-2516.2011.02679.x 22044692

[32] Wei W Misra S Cannon MV Yang R Zhu X Gilmore R Molecular mechanisms of missense mutations that generate ectopic N-glycosylation sites in coagulation factor VIII. *Biochem J.* (2018) 475:873–86. 10.1042/BCJ20170884 29444815 PMC5957299

[33] Zheng C Liu H Zhou J Zhang B. EF-hand domains of MCFD2 mediate interactions with both LMAN1 and coagulation factor V or VIII. *Blood.* (2010) 115:1081–7. 10.1182/blood-2009-09-241877 20007547 PMC2817634

[34] Cunningham MA Pipe SW Zhang B Hauri H-P Ginsburg D Kaufman RJ. LMAN1 is a molecular chaperone for the secretion of coagulation factor VIII. *J Thromb Haemost.* (2003) 1:2360–7. 10.1046/j.1538-7836.2003.00415.x 14629470

[35] Swystun LL Notley C Georgescu I Lai JD Nesbitt K James PD The endothelial lectin clearance receptor CLEC4M binds and internalizes factor VIII in a VWF-dependent and independent manner. *J Thromb Haemost.* (2019) 17:681–94. 10.1111/jth.14404 30740857 PMC7083068

[36] O’Sullivan JM Jenkins PV Rawley O Gegenbauer K Chion A Lavin M Galectin-1 and Galectin-3 constitute novel-binding partners for Factor VIII. *Arterioscler Thromb Vasc Biol.* (2016) 36:855–63. 10.1161/ATVBAHA.115.306915 27013611

[37] Lee LY Lin C-H Fanayan S Packer NH Thaysen-Andersen M. Differential site accessibility mechanistically explains subcellular-specific N-glycosylation determinants. *Front Immunol.* (2014) 5:404. 10.3389/fimmu.2014.00404 25202310 PMC4142333

[38] He L-Z Crocker A Lee J Mendoza-Ramirez J Wang X-T Vitale LA Antigenic targeting of the human mannose receptor induces tumor immunity. *J Immunol.* (2007) 178:6259–67. 10.4049/jimmunol.178.10.6259 17475854

[39] Ahlén G Strindelius L Johansson T Nilsson A Chatzissavidou N Sjöblom M Mannosylated mucin-type immunoglobulin fusion proteins enhance antigen-specific antibody and T lymphocyte responses. *PLoS One.* (2012) 7:e46959. 10.1371/journal.pone.0046959 23071675 PMC3470573

[40] Geijtenbeek TBH van Vliet SJ Engering A’t Hart BA van Kooyk Y. Self- and nonself-recognition by C-type lectins on dendritic cells. *Annu Rev Immunol.* (2004) 22:33–54. 10.1146/annurev.immunol.22.012703.104558 15032573

[41] Martinez-Pomares L. The mannose receptor. *J Leukoc Biol.* (2012) 92:1177–86. 10.1189/jlb.0512231 22966131

[42] Lundberg K Albrekt A-S Nelissen I Santegoets S de Gruijl TD Gibbs S Transcriptional profiling of human dendritic cell populations and models–unique profiles of in vitro dendritic cells and implications on functionality and applicability. *PLoS One.* (2013) 8:e52875. 10.1371/journal.pone.0052875 23341914 PMC3544800

[43] Dasgupta S Repessé Y Bayry J Navarrete A-M Wootla B Delignat S VWF protects FVIII from endocytosis by dendritic cells and subsequent presentation to immune effectors. *Blood.* (2007) 109:610–2. 10.1182/blood-2006-05-022756 16985172

[44] Linehan SA Martínez-Pomares L Stahl PD Gordon S. Mannose receptor and its putative ligands in normal murine lymphoid and nonlymphoid organs: in situ expression of mannose receptor by selected macrophages, endothelial cells, perivascular microglia, and mesangial cells, but not dendritic cells. *J Exp Med.* (1999) 189:1961–72. 10377192 10.1084/jem.189.12.1961 PMC2192961

[45] Burgdorf S Lukacs-Kornek V Kurts C. The mannose receptor mediates uptake of soluble but not of cell-associated antigen for cross-presentation. *J Immunol.* (2006) 176:6770–6. 10.4049/jimmunol.176.11.6770 16709836

[46] Martinez-Pomares L Hanitsch LG Stillion R Keshav S Gordon S. Expression of mannose receptor and ligands for its cysteine-rich domain in venous sinuses of human spleen. *Lab Invest.* (2005) 85:1238–49. 10.1038/labinvest.3700327 16056240

[47] Pack M Trumpfheller C Thomas D Park CG Granelli-Piperno A Münz C DEC-205/CD205+ dendritic cells are abundant in the white pulp of the human spleen, including the border region between the red and white pulp. *Immunology.* (2008) 123:438–46. 10.1111/j.1365-2567.2007.02710.x 17944899 PMC2433334

[48] Lech M Susanti HE Römmele C Gröbmayr R Günthner R Anders H-J. Quantitative expression of C-type lectin receptors in humans and mice. *Int J Mol Sci.* (2012) 13:10113–31. 10.3390/ijms130810113 22949850 PMC3431848

[49] Navarrete A Dasgupta S Delignat S Caligiuri G Christophe OD Bayry J Splenic marginal zone antigen-presenting cells are critical for the primary allo-immune response to therapeutic factor VIII in hemophilia A. *J Thromb Haemost.* (2009) 7:1816–23. 10.1111/j.1538-7836.2009.03571.x 19682235

[50] Delignat-Heudier S. *Therapeutic Strategies Against FVIII Immune Response in Hemophilia A?: By Modifying FVIII Structure, by Inhibiting B Cells Signalisation.* (2017). Available online at: https://tel.archives-ouvertes.fr/tel-01537891 (accessed November 28, 2019).

