# Supplementary material for: Removal of Mannose-Ending Glycan at Asn2118 Abrogates FVIII Presentation by Human Monocyte-Derived Dendritic Cells
Source: Front Immunol. 2020 Mar 26;11:393. doi: 10.3389/fimmu.2020.00393 (PMC7117063; doi:10.3389/fimmu.2020.00393)
Supplement: Supplementary file 1 [file Data_Sheet_1.docx]

**Supplemental information**

**Material and Methods**

**Generation of the FVIII-specific mouse T-cell hybridoma 1G8-A2**

The 1G8-A2 hybridoma was generated by immunizing Sure-L1 mice (HLA-A2.1-/HLA-DR1-transgenic H-2 class I-/class II-knockout mice), with 140µg of BDD-FVIII (Refacto, Pfizer) in complete Freund’s adjuvant, and fifteen days later with 100µg of recombinant C2 in incomplete Freund’s adjuvant. Four days later, cells of lymph nodes were collected and stimulated 72 hr with BDD-FVIII. Purified T cells were then fused with BWZ36 (TCR^-/-^) fusion partner cells using polyethylene glycol. Screening of FVIII-specific T-cell hybridomas was performed using BDD-FVIII and splenocytes from SureL1 mice (as a source of APCs), upon quantification of the secreted Il-2 by ELISA. The clone 1G8-A2 was obtained after 3 sub-clonings by limiting dilutions.

**Figures**

**
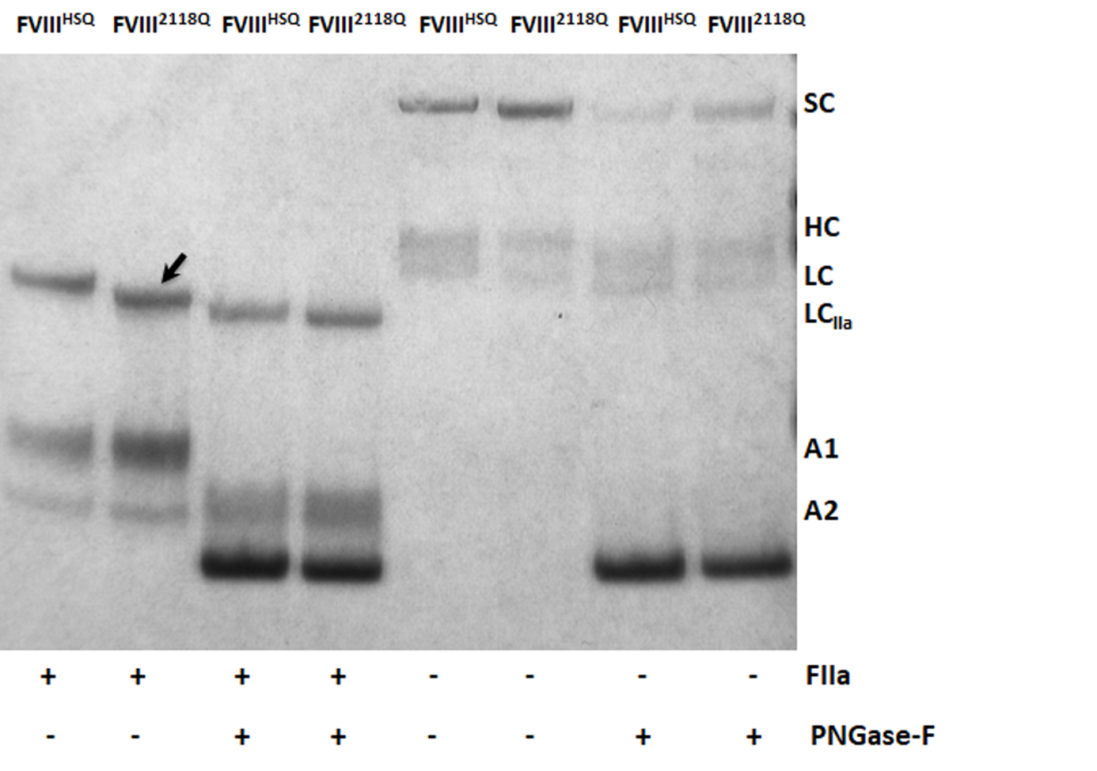
**

**Supplemental Figure 1:** SDS-PAGE of FVIII^HSQ^ and FVIII^2118Q^ with and without exposure to thrombin (FIIa) and peptide N-glycosidase f (PNGase-F). FVIII^HSQ^ and FVIII^2118Q^ were exposed or not to FIIa followed by denaturation and incubation alone or with PNGase-F. A silver staining of the gel was performed to detect proteins. SC, single chain FVIII; HC, FVIII heavy chain (A1-A2 domains); LC, FVIII light chain; LC_IIa_, thrombin-cleaved light chain; A1, A1 domain; A2, A2 domain. The dark band below A2 corresponds to FIIa and PNGase.


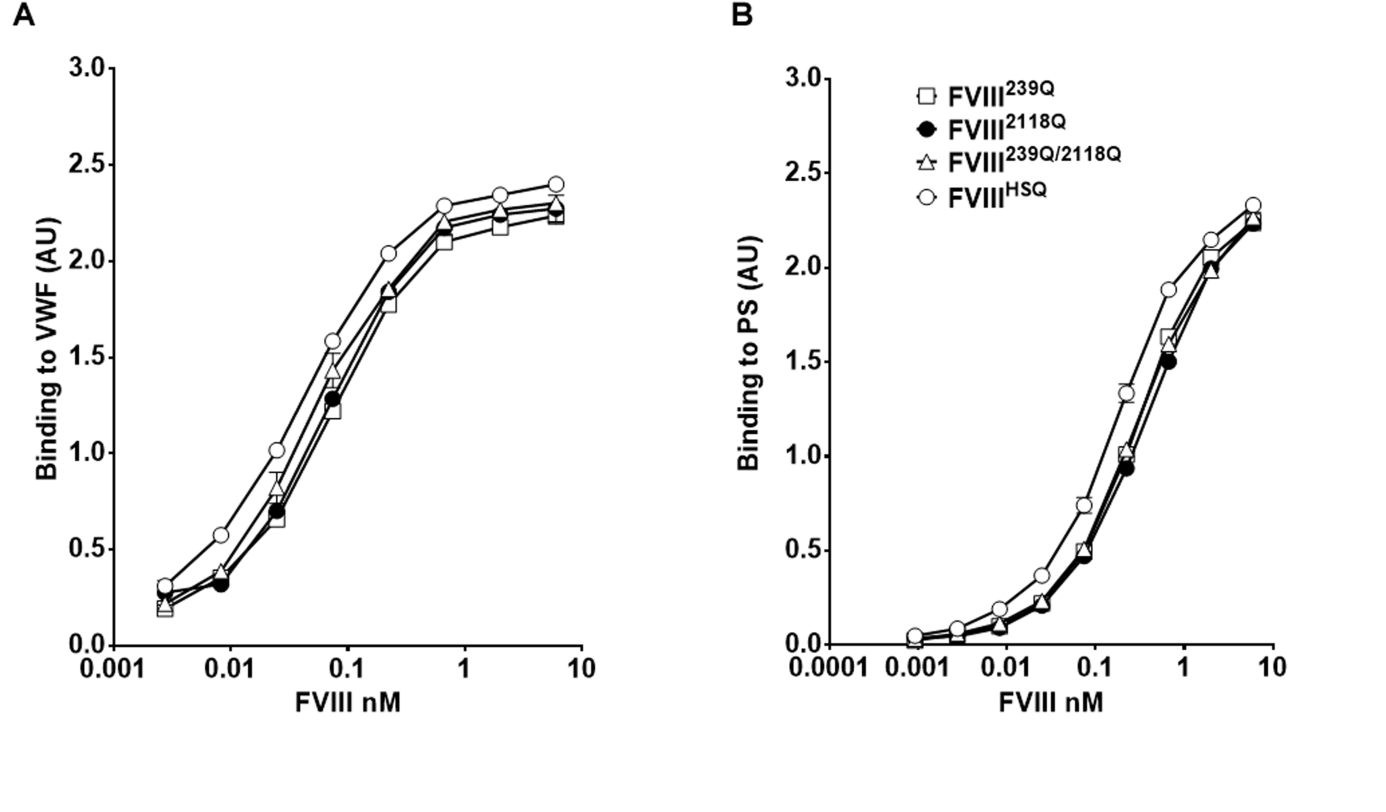


**Supplemental Figure 2.** Panel A and B. Elisa plates were coated with human von Willebrand factor (VWF, A), or 1,2-Diacyl-sn-glycero-3-phospho-L-serine (PS, B). The different recombinant B-domain deleted FVIII were incubated at different concentrations and revealed with a biotinylated anti-human FVIII antibody (GMA-8015), streptavidin-HRP and the peroxidase substrate. The binding of FVIII is depicted as the optical density measured at 492 nm in arbitrary units (AU).


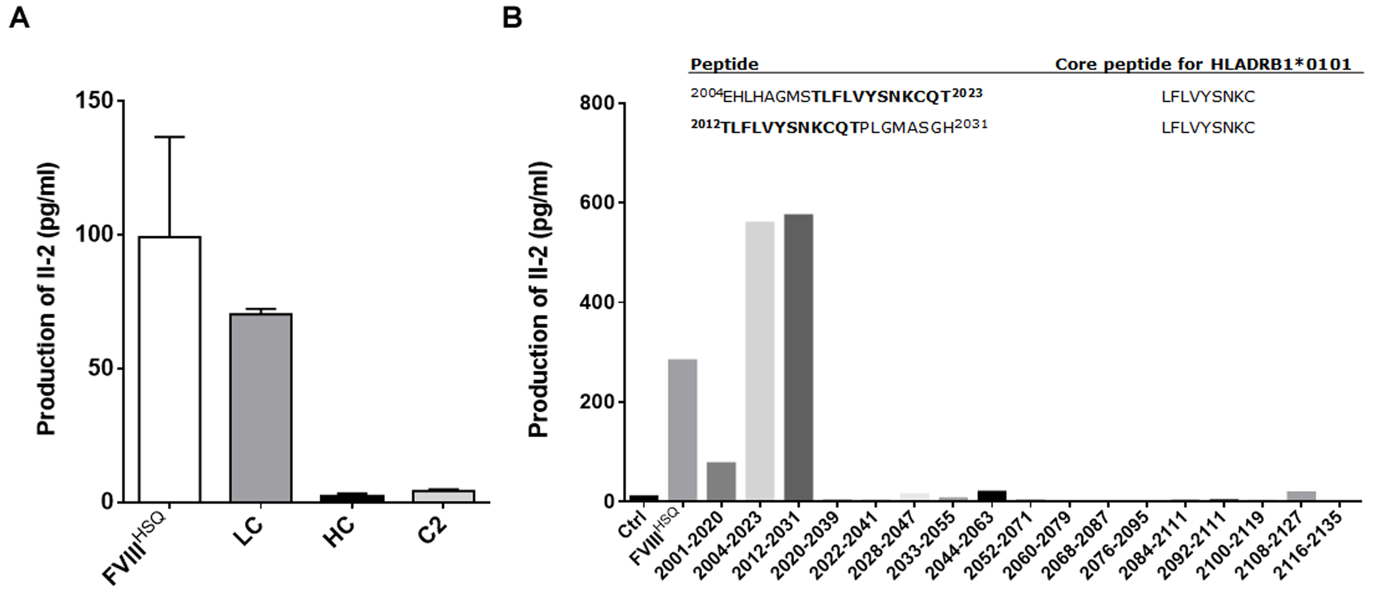


**Supplemental Figure 3. Screening of FVIII peptide specificity of T cell hybridoma 1G8-A2.** Specificity of the FVIII-specific HLA-DRB1*0101-restricted T cell hybridoma 1G8-A2 was screened against light chain (LC), heavy chain (HC) and C2 domain of FVIII (C2) by FVIII-loaded HLA-matched human MO-DCs (Panel A). Overlapping peptide of the C1 domain were further tested with loaded HLA-matched human MO-DCs (Panel B). Table 1. Prediction of peptides binding to HLA DRB1*0101 with IEDB database shows that C1.1 and C1.3 sequence share only one common core peptide.





**Supplemental Figure 4.** **Removal of both N239 and N2118 glycosylation sites do not have an additional effect on T-cell activation by human MO-DCs.** The activation of 1G8-A2 by FVIII-loaded (10 nM) HLA-matched human MO-DCs was evaluated. IL-2 produced by the activated T cells was measured in the supernatant after 24 hr. Representative of two independent experiments (mean±SEM).
